# Resurgence risk for malaria, and the characterization of a recent outbreak in an Amazonian border area between French Guiana and Brazil

**DOI:** 10.1186/s12879-020-05086-4

**Published:** 2020-05-26

**Authors:** Emilie Mosnier, Isabelle Dusfour, Guillaume Lacour, Raphael Saldanha, Amandine Guidez, Margarete S. Gomes, Alice Sanna, Yanouk Epelboin, Johana Restrepo, Damien Davy, Magalie Demar, Félix Djossou, Maylis Douine, Vanessa Ardillon, Mathieu Nacher, Lise Musset, Emmanuel Roux

**Affiliations:** 1 Unité des Maladies Infectieuses et Tropicales, Centre Hospitalier Andrée Rosemon, rue des flamboyants, 97306 Cayenne, French Guiana; 2 Aix Marseille University, INSERM, IRD, SESSTIM, Sciences Economiques & Sociales de la Santé & Traitement de l’Information Médicale, Marseille, France; 3 Unité Contrôle et Adaptation des Vecteurs, Institut Pasteur de la Guyane, 23 avenue Pasteur, 97306 Cayenne, French Guiana; 4 Altopictus, 67 avenue Maréchal Juin, 64200 Biarritz, France; 5 LIS, ICICT, Fiocruz, Av. Brasil, 4365, Manguinhos, Rio de Janeiro, Brazil; International Join Laboratory Sentinela, IRD, Fiocruz, University of Brasília, Rio de Janeiro, Brazil; 6 Superintendência de Vigilância em Saúde do Amapá (SVS-AP), Av. 13 de Setembro, 1889 – Buritizal, Macapá, Amapá, Brazil; 7 Agence Régionale de la Santé, 66 rue des flamboyants, 97306 Cayenne, French Guiana; 8 Service d’Entomologie de la Direction Démoustication et Actions Sanitaires, collectivité Térritoriale de Guyane, carrefour de suzini 4179 route de montabo, 97307 Cayenne, French Guiana; 9 LEEISA (Laboratoire Ecologie, Evolution, Interactions des Systèmes Amazoniens), CNRS, Université de Guyane, IFREMER, 275 route de Montabo, 97300 Cayenne, France; 10 Laboratoire de Parasitologie et Mycologie, Centre Hospitalier Andrée Rosemon, rue des flamboyants, 97306 Cayenne, French Guiana; 11 Université de Guyane, EA3593 Ecosystèmes Amazoniens et Pathologie Tropicale, Cayenne, French Guiana; 12 Centre d’Investigation Clinique Antilles Guyane – Inserm 1424, Centre Hospitalier Andrée Rosemon, rue des flamboyants, 97306 Cayenne, French Guiana; 13 CIRE Guyane, 66 rue des flamboyants, 97306 Cayenne, French Guiana; 14 Laboratoire de parasitologie, Centre National de Référence du Paludisme, Pôle Zones Endémiques, WHO Collaborating Center for Surveillance of Antimalarial Drug Resistance, Institut Pasteur de la Guyane, 23 avenue Pasteur, 97306 Cayenne, French Guiana; 15 ESPACE-DEV, IRD, Universités de Montpellier, de La Réunion, de la Guyane, des Antilles, Montpellier, France: LIS, ICICT, Fiocruz, Av. Brasil, 4365, Manguinhos, Rio de Janeiro, Brazil; 16 International Join Laboratory Sentinela, IRD, Fiocruz, University of Brasília, Rio de Janeiro, Brazil

**Keywords:** *Plasmodium vivax*, *Anopheles darlingi*, French Guiana, Brazil, Transnational, Outbreak investigation, Indigenous south Americans, Malaria, Amazonia

## Abstract

**Background:**

In 2017, inhabitants along the border between French Guiana and Brazil were affected by a malaria outbreak primarily due to *Plasmodium vivax* (*Pv*). While malaria cases have steadily declined between 2005 and 2016 in this Amazonian region, a resurgence was observed in 2017.

**Methods:**

Two investigations were performed according to different spatial scales and information details: (1) a local study on the French Guiana border, which enabled a thorough investigation of malaria cases treated at a local village health center and the entomological circumstances in the most affected neighborhood, and (2) a regional and cross-border study, which enabled exploration of the regional spatiotemporal epidemic dynamic. Number and location of malaria cases were estimated using French and Brazilian surveillance systems.

**Results:**

On the French Guianese side of the border in Saint-Georges de l’Oyapock, the attack rate was 5.5% (*n* = 4000), reaching 51.4% (*n* = 175) in one Indigenous neighborhood. Entomological findings suggest a peak of *Anopheles darlingi* density in August and September. Two female *An. darlingi* (*n* = 1104, 0.18%) were found to be *Pv*-positive during this peak. During the same period, aggregated data from passive surveillance conducted by Brazilian and French Guianese border health centers identified 1566 cases of *Pv* infection. Temporal distribution during the 2007–2018 period displayed seasonal patterns with a peak in November 2017. Four clusters were identified among epidemic profiles of cross-border area localities. All localities of the first two clusters were Brazilian. The localization of the first cluster suggests an onset of the outbreak in an Indigenous reservation, subsequently expanding to French Indigenous neighborhoods and non-Native communities.

**Conclusions:**

The current findings demonstrate a potential increase in malaria cases in an area with otherwise declining numbers. This is a transborder region where human mobility and remote populations challenge malaria control programs. This investigation illustrates the importance of international border surveillance and collaboration for malaria control, particularly in Indigenous villages and mobile populations.

## Background

After two decades of global decreases in malaria incidence, rates are increasing for the first time since 2016 [1]. In the Americas, the largest increase was recorded in Brazil and Venezuela [1]. Thus, malaria remains a public health challenge in South America. In Brazil, transmission is mainly entrenched in the Amazon Basin, which accounts for 99.5% of Brazil’s malaria burden [2].

French Guiana is a European overseas, malaria-endemic territory. The Oyapock river forms the border between French Guiana and Brazil. This area has engaged in a regional malaria control program [3] which faces challenges concerning illegal populations of gold miners living in remote areas along the borders with Suriname and Brazil [2–5]. Malaria control interventions are carried out through the free distribution of insecticide-treated nets and access to artemisinin-based therapy on both sides of the border [3]. Along the Oyapock river, *P. falciparum* has declined over the past several years and *P. vivax* is now responsible for most malaria cases [6, 7]. Although transmission decline is observed on a global scale, local heterogeneities persist and must be addressed through targeted control initiatives in order to achieve malaria elimination [8, 9]. This is particularly important in border areas, which complicate effective implementation of malaria control interventions [2, 10]. *An. darlingi* is the predominant malaria vector species in this region [11, 12].

The objective of this article is to describe the 2017 malaria outbreak through an entomo-epidemiological investigation of the Saint-Georges de l’Oyapock (STG) region along the Oyapock river. This investigation is associated with a retrospective study using a spatiotemporal analysis of malaria case data from surveillance health systems of French Guiana and Brazil.

### Outbreak detection

The malaria surveillance system in French Guiana is based on three data sources: (1) Delocalized Centers for Prevention and Care (CDPS) reports, (2) laboratory services notification, and (3) military health services notification. Since the end of May 2017, data from the CDPS of STG showed a slow and persistent increase of *P. vivax* cases in the Indigenous neighborhood of Trois Palétuviers. These clusters and the subsequent local outbreak have no apparent link to any other transmission sites in French Guiana, nor with the epidemiological situation in the municipality of Oiapoque, an immediate neighbor on the Brazilian side of the border. Between September and October of 2017, increases in malaria cases were observed in downtown STG, French Guiana. This number largely exceeded the expected case number over the past 3 years for this endemic area [7].

Retrospectively, the data provided by the Brazilian malaria epidemiological surveillance information system (Sistema de Informação de Vigilãncia Epidemiológica: SIVEP-Malária) revealed an increase in the number of cases of malaria infections in the Oiapoque municipality, including among the Uaçá, Galibi and Juminã indigenous territories (Amapá, Brazil).

## Methods

### Study area and population

**Fig. 1:**
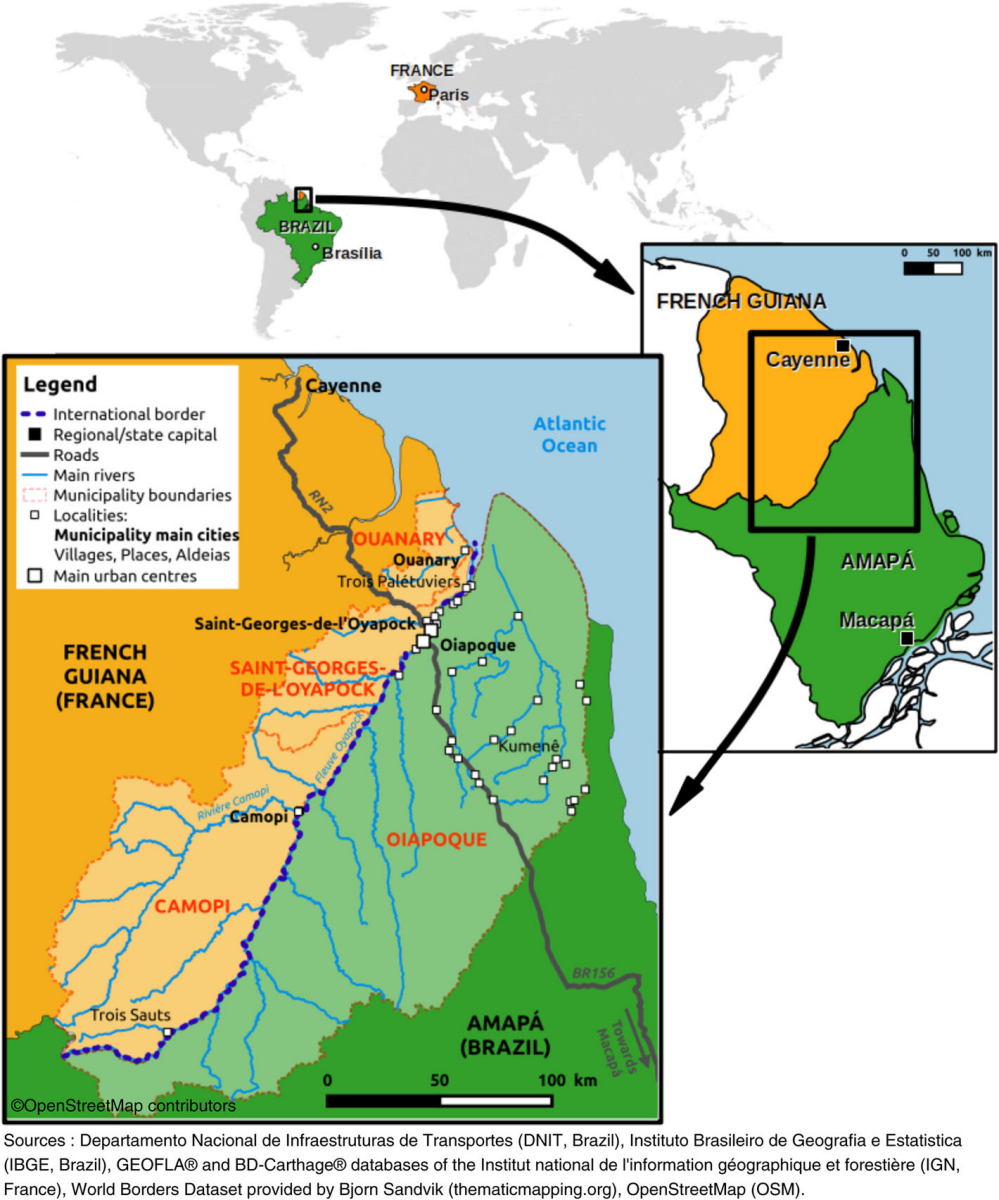
Map of the border area between Brazil and French Guiana. (Map data comes from Departamento Nacional de Infraestruturas de Transportes (DNIT, Brazil), Instituto Brasileiro de Geografia e Estatistica (IBGE, Brazil), GEOFLA® and BD-Carthage® databases of the Institut national de l’information géographique et forestière (IGN, France), World Borders Dataset provided by Bjorn Sandvik (thematicmapping.org), OpenStreetMap (OSM)). Map was created by using QGIS Geographic Information System. Open Source Geospatial Foundation Project. http://qgis.osgeo.org

On the French Guianese side, the border region of Oyapock includes three municipalities: Camopi, STG and Ouanary, with approximately 1700, 4000 and 100 residents, respectively, in 2017, according to the STG health center census. The region marks the northeastern border between French Guiana and Brazil (Fig. 1). On the Brazilian side, the frontier region includes the Brazilian municipality of Oiapoque, which includes 25,514 inhabitants according to a 2015 estimation by the Brazilian National Institute of Statistics (IBGE), and lies along the Oyapock river (Fig. 1). To the north and east of the Oiapoque municipality, there are a large number of small villages. Indigenous territories (Uaçá, Galibi, Juminã) predominate. This border region consists of a vast, remote territory of Amazonian rainforest, associated with highly variable population densities. There is a great diversity of populations, including Indigenous South Americans (mainly Wayãpi, Teko, Palikur, Karipuna, Galibi-Marworno and Galibi peoples), Creoles, Saramaka, and migrants from other Brazilian states who migrated to the area to work in the gold mining sector (mainly in illicit gold mining or border supply zones) [13]. Daily transborder exchanges exist among these populations [13]. The climate is equatorial, with four alternating seasons: a long rainy season from April to June, a dry season from July to December, a short, rainy season from January to February and a short, dry season in March. The mean annual temperature is 25.9 °C and the annual rainfall is ~ 3405 mm [14]. The cities of STG and Oiapoque are persistent, low-malaria endemicity areas [7].

### Malaria case definition

Malaria diagnosis was performed in health centers using the Rapid Diagnosis Test (RDT) - SD Bioline® Malaria Ag *Pf/Pan* in French Guiana, and thick and thin smears or RDTs were used in Brazil. A malaria case was defined as a patient with RDT or microscopy-positive results. Data included passive monitoring of cases in border health centers in both French Guiana and Brazil, and also active case detection around positive cases in the city of Oiapoque.

Before treatment with primaquine, a glucose-6-phosphate dehydrogenase (G6PD) deficiency test (Roche diagnostics®, instrumentation Cobas 6000) was conducted at least 2 weeks after the malaria infection in French Guiana [15].

In French Guiana, *P. vivax* relapse was defined as having a medical history of malaria within a period of 7 to 90 days since last malaria diagnosis. This interval was considered adequate in length to distinguish follow-up (0–7 days), relapse (8–90 days) and new infection (> 90 days) [7, 16].

In Brazil, the absence of a unique patient identification code did not permit the use of the same relapse identification method. Nevertheless, malaria attacks related to patient follow-up, treatment failures, and potential relapses were identified during the medical consultation and denoted as *treatment verification slide* (Lâmina de Verificação de Cura, “LVC”) in the database. A malaria attack was considered an LVC for *P. vivax* if the patient was positive for *P. vivax* and received a treatment against *P. vivax* malaria during the last 60 days. It is worth noting that *P. vivax* relapses are expected to be less likely to occur in Brazil due to a systematic primaquine administration (except for specific cases such as pregnancy), which do not exist on the French Guianese side.

As listed below, two investigations were performed according to different spatial scales and information details:

i) a local study on the French Guiana border, which enabled a thorough investigation of malaria cases treated at a local village health center and the entomological circumstances in the most affected neighborhood a regional and cross-border study, which enabled exploration of the regional spatiotemporal epidemic dynamic.
ii) a regional and cross-border study, which enabled exploration of the regional spatiotemporal epidemic dynamic.

### Local investigations on the French Guianese side

#### Epidemiological description

Medical record data from the STG health center allowed for a retrospective analysis of epidemics. Malaria cases diagnosed between January 1, 2017 and January 31, 2018 were included, and the following variables were analyzed: (1) age, (2) gender, and (3) outcome/location of acute *P. vivax* cases (and relapses) treated in the STG health center [17]. Census data were retrieved from the STG health center to calculate the incidence and attack ratio by neighborhood (~ 4000 inhabitants). Factors associated with risk of attack/relapse were identified by univariate analysis. A chi-square test was used for nominal data, a student’s t-test was applied when the test statistic followed a normal distribution, and a Mann-Whitney U test was used for skewed data.

#### Entomological investigations

The entomological investigation focused on Trois-Palétuviers, a neighborhood of STG where the greatest incidence of malaria cases occurred during this period, according to the local health center team. Mosquitoes were collected monthly from August through November 2017 over 2 to 3 consecutive nights per month. Two octenol-baited Mosquito Magnet® traps were used to collect anopheline species and were supplemented with BG-Sentinel and Center for Disease Control and prevention (CDC) light traps [18]. The collections were performed periodically from 18:00 to 07:00. Intra-domiciliary aspirations were done inside four houses between 19:00 and 20:00 in August. Mosquito species were identified morphologically by entomologists of Pasteur Institute of French Guiana according to identification keys specifically adapted to the Anopheline species present in the region [19].

The infectious statuses of the Anopheles specimens captured from August through October were investigated. The head and thorax of 1218 females were dissected and placed in an agitator with grinding beads. The DNA of each sample was extracted using a Magjet Genomic DNA kit (Thermo Scientific, K2722), then 10 female DNA samples were pooled for polymerase chain reaction (PCR). The presence of *P. falciparum*, *P. vivax* and *P. malariae* parasites was investigated using a nested PCR method according to Snounou et al. [20]. Individual confirmation was performed for each positive pool.

#### Meteorological data

The meteorological influence on malaria cases and vector abundance was investigated as the development of the vector *An. darlingi* has been correlated with monthly rainfalls in the region [21]. The average air temperatures and daily precipitation were obtained from the STG meteorological station (Météo France n° 97,308,001) [22].

### Regional analysis of the cross-border epidemic dynamic

#### Registries

French Guianese data came from border area health centers (Delocalized Centers for Prevention and Care CDPS).

Brazilian data came from the Brazilian information system dedicated to epidemiological surveillance (Sistema de Informações de Vigilância Epidemiológica da Malária, SIVEP-Malária).

International cooperation, research and technological development works have been conducted for several years and led to the establishment of an operational system for the harmonization and visualization of data from the two aforementioned databases [23]. This system relies on expert knowledge, meets international standards for information representation, and uses dedicated and robust harmonization techniques and tools (notably the Extract, Transform and Load, “ETL”, approach). It enables the temporospatial monitoring of the epidemiological situation of cross-border malaria between French Guiana and Brazil.

#### Epidemic profile clustering

In order to describe the epidemic dynamics in the study area, the epidemic profiles of cross-border area localities were defined and clustered, and the resulting clusters were represented and interpreted both temporally and spatially. To achieve this, we first selected localities presenting a significant number of cases, by considering each country individually. The employed method included the following:

i) ranking all localities according to their total number of cases during the study period
ii) selecting all localities that contributed up to 90% of the total number of cases. This was done by first ranking the localities in descending order by number of cases and calculating the cumulative number of cases as a percentage of the total. This allowed us to consider epidemic profiles only for localities with a significant number of cases, while ensuring the representativeness of the dataset. Time-series of malaria cases were obtained by aggregating the daily case counts on a weekly basis. Locality epidemic profiles were defined by the normalized cumulated numbers of malaria cases. This method highlighted the curves dynamics and facilitated their interpretation.

Next, Ward’s hierarchical clustering method using Euclidean distance was applied to the epidemic profiles.

#### Ethical approval

The French Guianese database was anonymized and declared to the *Commission Nationale Informatique et Libertés* (CNIL) (authorization N° 1,939,018). Brazil’s surveillance registries database was anonymized prior to being sent. The cross-border malaria information system (Saldanha et al., submitted) was also approved by the CNIL (N°2,135,463). All of the actions carried out in Brazilian malaria registries are authorized as part of Fiocruz public health activities, as per the Brazilian “free access” law 12.527 of November 18, 2011 and in compliance with law 13.709, of August 14, 2018.

## Results

### Epidemiological description of malaria cases within the French Guianese border area (STG health center)

**Fig. 2:**
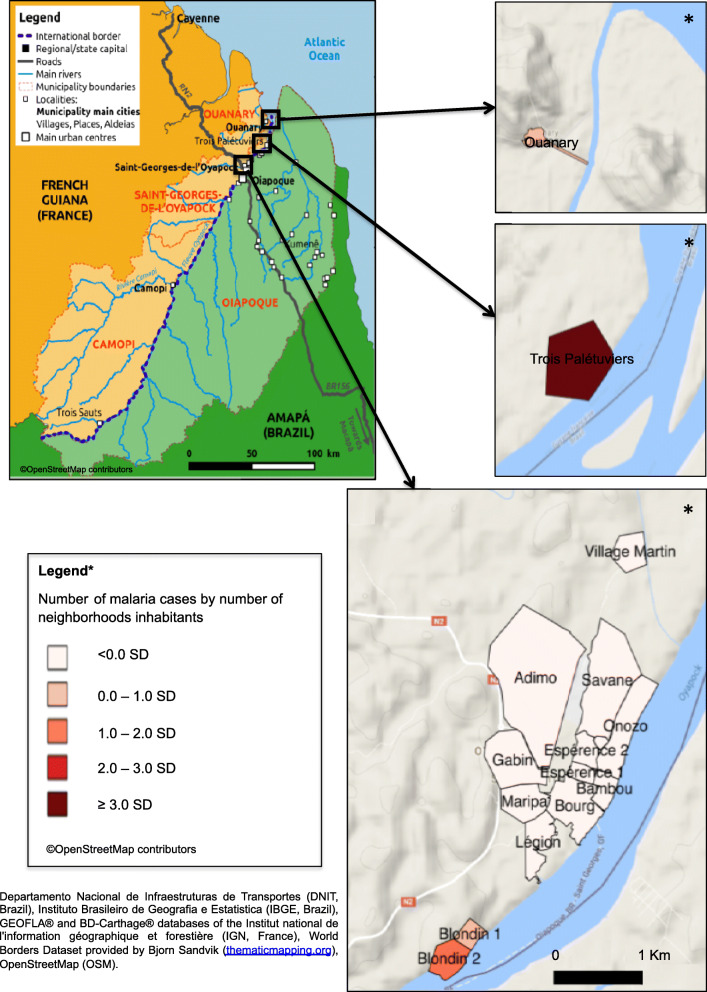
Mapping the incidence of *P. vivax* cases in neighborhood inhabitants of the French Guianese border area (Saint-Georges de l’Oyapock and Ouanary villages), January 2017–January 2018. (The map data comes from Departamento Nacional de Infraestruturas de Transportes (DNIT, Brazil), Instituto Brasileiro de Geografia e Estatistica (IBGE, Brazil), GEOFLA® and BD-Carthage® databases of the Institut national de l’information géographique et forestière (IGN, France), World Borders Dataset provided by Bjorn Sandvik (thematicmapping.org), OpenStreetMap (OSM)). Map was created by using QGIS Geographic Information System. Open Source Geospatial Foundation Project. http://qgis.osgeo.org

During the study period from January 1, 2017 to January 31, 2018, 219 people were infected with *P. vivax* (primary attack). The median age was 22 years [interquartile range (IQR) = 19.9–24.27]; 50.7% (*n* = 219) of cases were less than 18 years old. The sex ratio (M:F) was 1.23 (*n* = 121:98). A significant portion (27%, *n* = 59) experienced at least one relapse of *P. vivax* infection, 6.9% (*n* = 15) experienced 2 relapses, and 1% (*n* = 3) experienced 3 relapses. The spatiotemporal distributions of cases suggest that since May 2017 the risk of infection steadily increased among the residents of STG, with a peak in November 2017. The attack rate was 5.5% in the STG municipality, reaching 51.4% in the Trois-Palétuviers neighborhood (Fig. 2). Children (< 18 years old) had a significantly greater risk of relapse *P* < 0.005, OR = 4.03 [2.00–8.31]). The risk of relapse was the same among both males and females. Residents of the Trois-Palétuviers neighborhood had a higher risk of relapse compared to other STG neighborhoods (*P* = 0.037).

Although 16.5% (*n* = 188) of patients had a G6PD activity below laboratory reference values (N: 10–14 U/HgL), only 6.9% (*n* = 188) of patients had an intermediate G6PD deficiency, and no severe deficiency was reported (Supplement S1). In January 2018, 68.5% (*n* = 150) of malaria cases received complete treatment (chloroquine and primaquine), 6.4% (*n* = 14) were only treated with chloroquine due to a contraindication for primaquine (pregnancy, breastfeeding or G6PD deficiency), 1.3% (*n* = 3) were treated in another health center, and 23.7% (*n* = 52) were lost in follow-up and treated only with chloroquine.

### Entomological findings

From August to November 2017, 1246 females of four *Anopheles* species were collected, and 97.4% of them were captured with Mosquito-Magnets. *An. darlingi* was the predominant Anopheline *s*pecies (*n* = 1230), representing 99.2% of those captured (excluding seven Anopheline specimens unidentified at the species level). Other species collected were *An. intermedius* (eight individuals in August), *An. nuneztovari* (one in September) and *An. oswaldoi* (one in October).

Of the 1218 *Anopheles* specimens tested for malaria by PCR, two female *An. darlingi* were found to be positive for *P. vivax.* One was captured on August 3rd (between 5:30 and 8:00) and another on September 13th (between 20:00 and 21:00). Both infected mosquitoes were captured in Mosquito-Magnets placed on the outskirts of the neighborhood, less than 50 m from the forest. Prevalence was estimated at 0.18% in August, and September 2017 (n_August_ = 514 and n_September_ = 590; *P* ≥ 0.05).

**Fig. 3:**
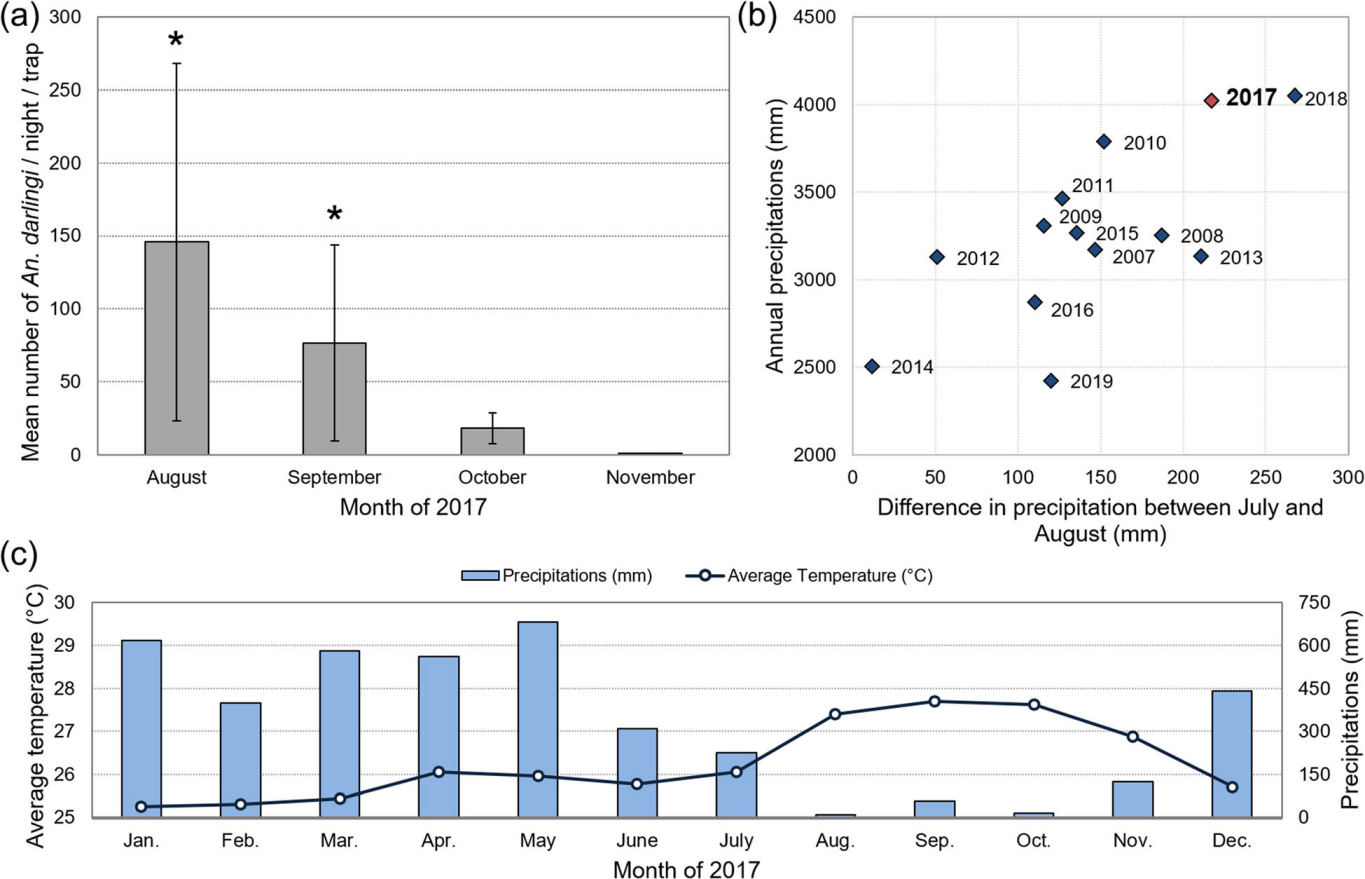
Vector abundance and climate data: (**a**) Monthly mean number of *An. darlingi* captured by night, Trois-Palétuviers, French Guiana, August—November 2017. An asterisk (*) represents the month(s) during which one mosquito infected with *P. vivax* was captured. Only the collection of Mosquito-Magnets in village outskirts were considered. (**b**) Annual precipitation and difference in monthly precipitation in the wet/dry season transition period (2011—2019) in Saint-Georges de l’Oyapock, and (**c**) Climatological data from the year 2017 (Source: Météo France)

The density of *An. darlingi* in the village outskirts was higher in August and September of 2017 than in October and November of 2017 (*P* = 0.045), with no significant peak in August (Fig. 3a). *An. darlingi* was captured at all hours of the collection period. It was also the only Anopheline species found to bite within households in the evening in August. The very high vector abundance monitored in August–September ran parallel to dry season months (Fig. 3b), and may be associated with the extreme seasons in 2017, with a rainy season and a dry season of uncommon magnitudes (Fig. 3c).

### Seasonality and a comparison of the French Guianese and Brazilian border areas from 2007 to 2017

**Fig. 4:**
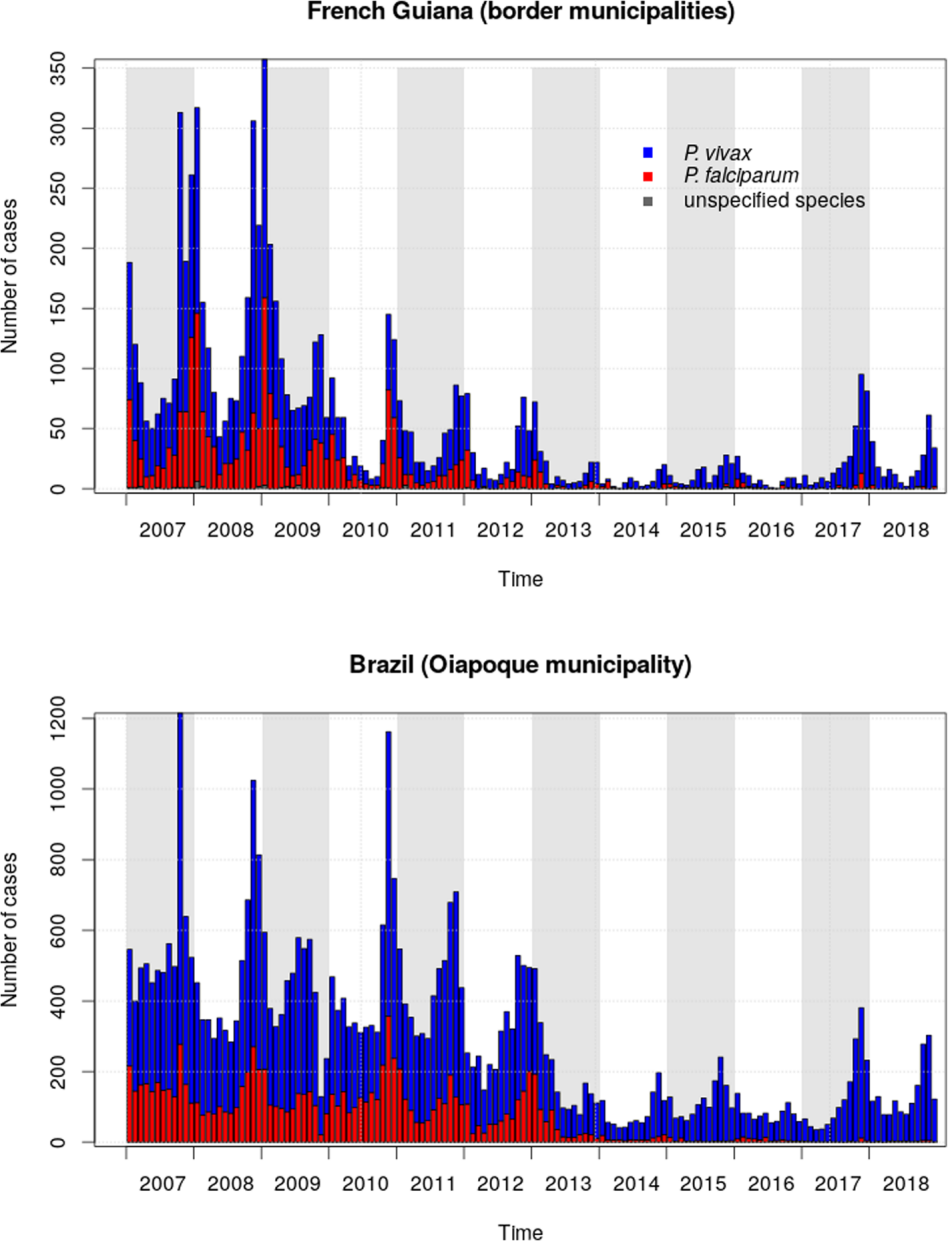
Monthly temporal dynamics of malaria cases in French Guiana and Brazil border areas

Malaria infection demonstrated a distinct seasonal variation on the French Guiana and Brazilian border with peaks in November (Fig. 4). Thus, most malaria cases in French Guiana (63.1%, *n* = 5385) occurred during the dry season (approximately July to December). The 2017 outbreak clearly demonstrated the same profile as previous epidemic cycles. In recent years, malaria infections have mostly been due to *P. vivax,* on both sides of the transnational border.

### Epidemic profile analysis

**Fig. 5:**
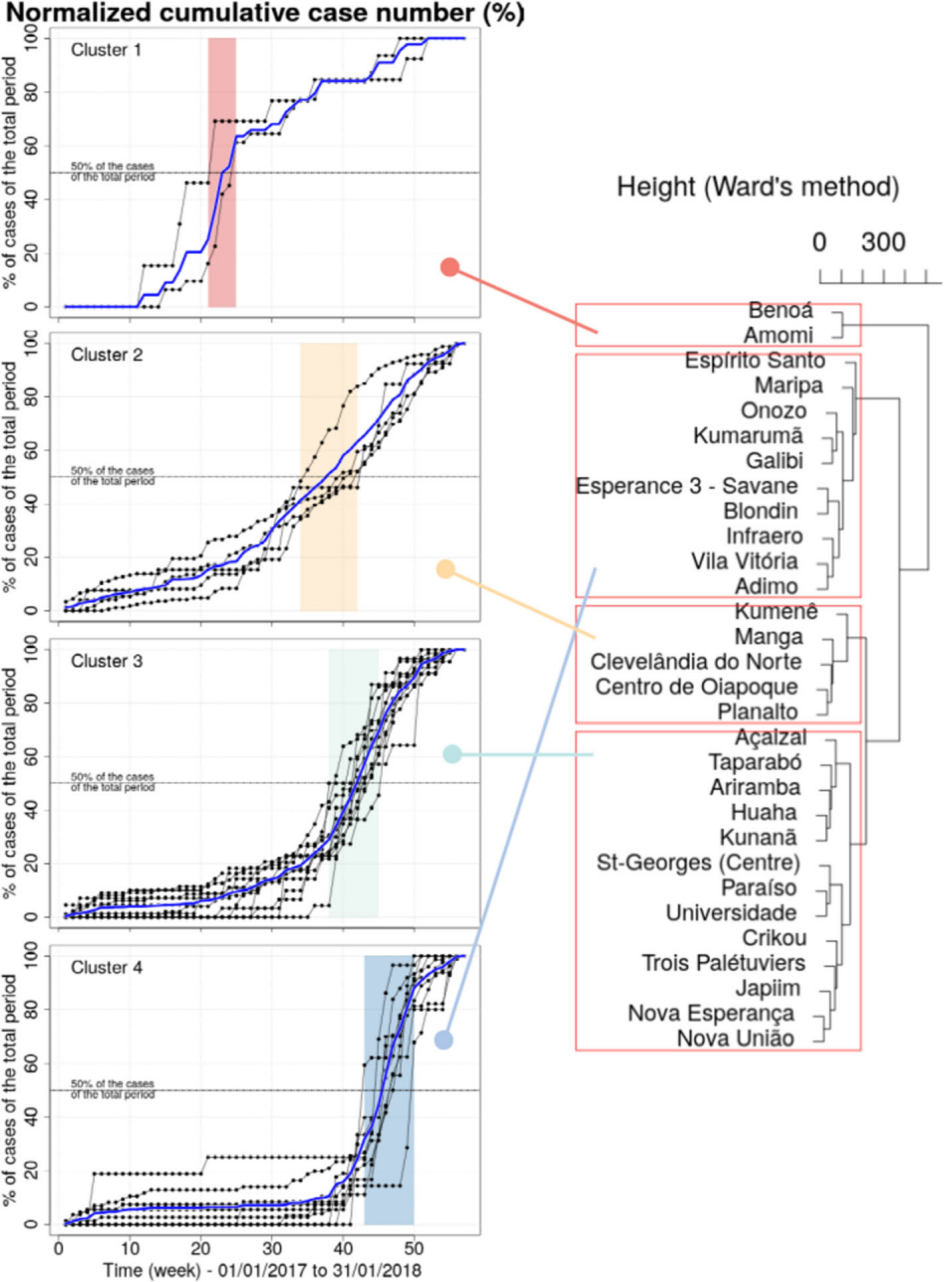
On the right: A dendrogram resulting from hierarchical clustering (using Euclidean distance and a Ward aggregation method). On the left: the normalized cumulative case numbers according to clusters on the cross-border region between French Guiana and Brazil, January 2017—January 2018. The period during which 50% of the total case number is reached, for all locality clusters, is represented in color. The blue curve represents the normalized cumulative curve of the total number of cases per cluster

**Fig. 6:**
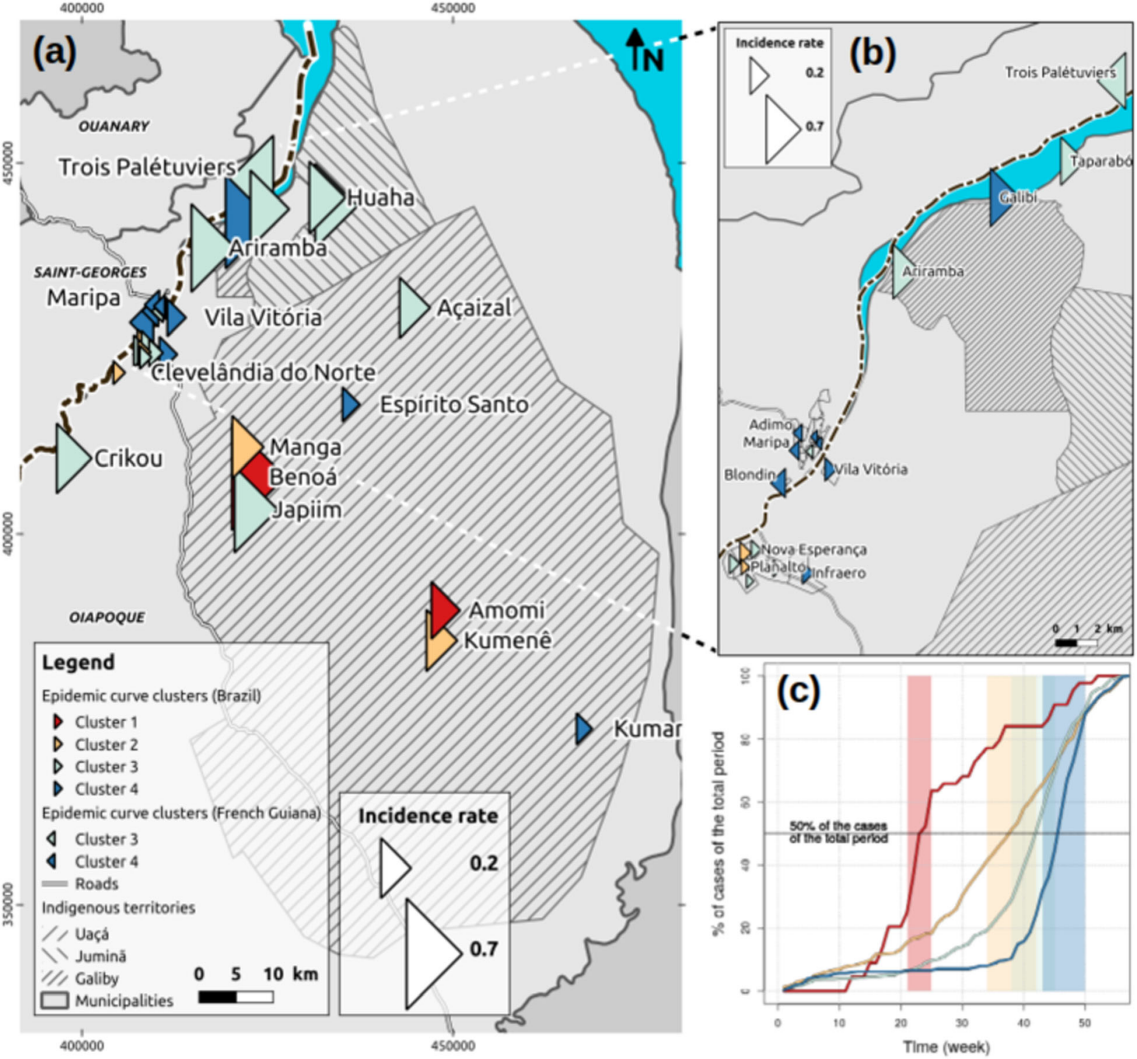
Choropleth of localities and clusters within the cross-border region between French Guiana and Brazil, January 2017—January 2018: (**a**) All cross-border localities included in the cluster analysis, (**b)** focus on the border, (**c**) normalized cumulative curves for the mean number of malaria cases per week and per cluster (same color code as in Fig. 1). The map data come from Departamento Nacional de Infraestruturas de Transportes (DNIT, Brazil),), Instituto Brasileiro de Geografia e Estatistica (IBGE, Brazil), Fundação Nacional do Índio, Brazil (FUNAI, Brazil) and GEOFLA® databases of the Institut national de l’information géographique et forestière (IGN, France). Map was created by using QGIS Geographic Information System. Open Source Geospatial Foundation Project. http://qgis.osgeo.org. Size of triangles is proportional to incidence rate. The color of triangle corresponds to the color code used in Fig. 1. Bottom right: normalized cumulative curves for the mean number of cases per cluster (same color code as in Fig. 1)

The total number of cases reported in registries from January 1, 2017 to January 31, 2018 was 1664 (1434 in Brazil, and 182 in French Guiana). Forty-eight cases were associated with unspecified localities of residence and were ignored (nine were notified in Brazil, with Brazil as the country of residence; the 39 remaining cases were notified in French Guiana without any mention of country of residence). Figure 5 illustrates the results of epidemic curve clustering. The dendrogram resulting from the hierarchical clustering clearly accentuates four clusters that correspond to low intra-cluster and high inter-cluster variances. Clusters were arranged chronologically by considering the time interval during which the weekly case numbers reached their maximum (corresponding to the inflection points of the cumulated curves). The inflection point corresponded approximately to the moment when the number of cases reached 50% of the total number of cases during the total considered period (Fig. 5). All localities of the first two clusters are Brazilian (Fig. 6 and Supplement S2). Fig. 6 is a map of the localities and clusters. All localities are situated in the northern part of the study area. Indigenous areas were reached first, demonstrating high incidence rates compared to the French STG or Brazilian Oiapoque city centers (Fig. 6). Cluster 1 included two Indigenous localities, with a much earlier start of the epidemic than elsewhere (between weeks 21–25) (Fig. 6 and Supplement S2). The second cluster included another two Indigenous villages (mostly Palikur inhabitants) close to the first cluster (Fig. 6 and Supplement S2). Cluster 3 aggregated French Guianese neighborhoods and the Oiapoque city center, including Trois-Palétuviers and Brazilian villages, which broadened the progressive spread of the outbreak (Fig. 6 and Supplement S2). Finally, cluster 4 represented STG and Oiapoque city centers and a continual expansion of the outbreak, in particular in the southeastern Kumarumã (Galibi-marworno) village (Fig. 6 and Supplement S2).

### Outbreak control measures

In French Guiana, the implemented control measures included the creation of a dedicated medical team with an infectious disease specialist physician, a Cayenne Hospital-based nurse, and cultural mediators from a local non-profit organization (Development, Animation, Accompanying measures, Cooperation: DAAC). This team was assembled in November 2017 and financed by the regional health agency of French Guiana and Cayenne Hospital. The team has contributed to improving the diagnosis and follow-up treatment of malaria in STG. The medical team used automobiles or canoes to directly visit households with malaria cases in order to expedite treatment, and control transmission. The team conducted malaria and G6PD blood testing, and provided comprehensive malaria treatment (chloroquine and primaquine). In order to facilitate outbreak control, the French National Agency for Medicines and Health Products Safety (ANSM) implemented a temporary accelerated treatment authorization procedure in order to quicken access to primaquine. Vector control measures were also deployed to reduce contact between people and Anopheles mosquitoes: long-lasting insecticidal nets (LLINs) were distributed to every malaria positive individual and their family members, as well as to the entire population of Trois-Palétuviers. Indoor residual spraying with deltamethrin was also conducted. Good practices to avoid mosquito “bites” were verbally explained to individuals and to the greater community in public spaces.

In Brazil, active case detection by thick and thin blood smears, along with indoor residual spraying were performed in Oiapoque city, as well as in Taparabú village (a Brazilian village facing the Trois-Palétuviers French Guianese neighborhood).

Since the beginning of the epidemic, transborder communication was established between the municipality of Oiapoque and the regional health agency of French Guiana in an aim to keep both parties informed of local trends, and to sustain preventive measures.

## Discussion

The peak of the 2017 malaria outbreak presented a greater intensity, however a similar pattern compared to previous seasons in both the French Guianese and Brazilian border areas. Several factors may explain this renewed increase in malaria infections. Firstly, there has been a shift from *P. falciparum* to *P. vivax* infections in this area over the last decade with greater difficulty (contraindication, availability of G6PD blood test and treatment) in treating with primaquine and thus in avoiding relapses [6, 24]. Secondly, increase in infection rates may be due to environmental causes: the abundance of *An. darlingi* in Trois-Palétuviers in August–September 2017—which was 7 to 14 times higher than the abundance previously reported from August–September 2013 and 2014 around STG [20] —may result from the magnitude of the rainy and dry seasons in 2017, local geological features and/or the impact of increased deforestation in Amazonia [22, 25]. Thirdly, a new municipal government was inaugurated in Oiapoque in 2017. With that, leadership roles shifted in the Special Indigenous Health District (DSEI), an entity responsible for providing health care to Indigenous peoples in Oiapoque. At the onset of this change in power dynamics, there is often an administrative discontinuity among local governments, which may have consequences for public services [26]. Finally, political and economic regional crises, particularly in Venezuela but also in Brazil, have led to mass human migration into Brazil and is associated with an increase of illicit gold mining [4, 27]. Unexpectedly, our results on incidence and spatial distribution revealed an additional human factor within the specific population of Indigenous South Americans. Study epidemic dynamics show that number of malaria cases began increasing in Indigenous Brazilian villages before increasing on the French Guianese side of the border. These communities were described in a recent study to be particularly at risk for malaria infection, and participated in factors driving this epidemic [5]. Remote Indigenous communities have specific behaviors, precarious living conditions, poor access to health care, higher mobility, as well as commercial, cultural or family links among populations from both sides of the border [5, 13]. This may explain the cross-border epidemic dynamic, and these communities represent a key population for malaria control in the region. Further investigations should be conducted to: (i) clearly identify these hypothetical mechanisms, (ii) confirm that such a scenario may have occurred in previous years, and (iii) show that observed events did not occur independently.

Indeed, studies of the transmission dynamics for each *Plasmodium* species genotype could help provide an overview of the spread of infection in this cross-border population.

In our study, a little less than 25% of patients (*n* = 219) were lost in follow-up after chloroquine treatment and didn’t undergo full treatment with primaquine. This result, however, does not change the main results concerning disease incidence. This is a common occurrence with *P. vivax* infection, specifically in Indigenous villages [24]. Indeed, people often feel better after chloroquine treatment and do not feel the need for additional treatment. Finally, in this study area, neighborhoods, which were more impacted by malaria, tended to be isolated. For the inhabitants of such remote areas, it is expensive and time-consuming to return to a given health center for primaquine treatment [5, 24].

Both contemporary and past studies have shown the significant influence of vector dynamics on malaria transmission [12, 28]. In 2017, the heaviest rainfall hit the area since 2000. This may have impacted the productivity of vector breeding sites in the transitional period between the wet and the dry season, and specifically the abundance of *An. darlingi* in the dry season [22]. In the specific study region, temperatures are usually not considered to be an accurate predictor of malaria vector presence and density, due to as it oscillates in the ideal range of values for the mosquito development all the time, and present diurnal variations that exceed the seasonal ones [29]. Past studies have demonstrated significant positive correlations between precipitation and *An. darlingi* abundance [12, 30]. Thus, we expect a significant relationship between precipitation and malaria case incidence. Between 2014 and 2019, during which case number stayed relatively stationary (Fig. 4), a short study performed by the authors showed that annual precipitation explained 91% (*r*^*2*^ = 0.91) of variance in the annual number of malaria cases in the STG and Oiapoque municipalities (in the framework of a univariate linear regression). In that sense, the 2017 recrudescence of cases could simply be considered a consequence of climate variability. The relationship between climatic variables and malaria incidence at a finer temporal scale is not trivial. Local ecological conditions [30], control actions, changes in population mobility patterns, etc., are all confounding factors. Eventually, beyond precipitation and temperature, many other climatological variables should be considered (humidity, radiation, etc.) (see for example Adde et al., 2016) [12]. As a consequence, estimating the relative impact of climate variability on malaria epidemiology is highly complicated, and beyond the strict scope of the present work.

Our study of *An. darlingi* confirm the well-known exo/endophagic behavior of this malaria vector, and the need to prevent host-vector contact inside of households (with mosquito nets) and outside of households (with the use of repellants and loose clothing). Several recent publications refer to insecticide resistance within this species in Brazil [31], and also an increased daytime activity [18]. These factors contribute to the challenge of vector control in the region. Because of the profusion of infected females in August and September—before the human peak in November—, *An. darlingi* was likely the main Anopheline species involved in the emergence of this outbreak. On the French Guiaese side of the border, most malaria cases occurred in neighborhoods with dense vegetation (Blondin and Trois-Palétuviers) where high densities of *An. darlingi* were reported both in past and contemporary studies [12, 21]. Blondin and Trois-Palétuviers are also the neighborhoods that are farthest from their proximal health center, 15 min and 45 min by canoe respectively. This isolation may contribute to the foregoing of medical treatment, and may explain the higher rate of relapse in the Indigenous neighborhood of Trois-Palétuviers. In considering the high attack rate in Trois-Palétuviers, our results suggest that the combination of vector density, exchanges and mobility among Indigenous Brazilian villages, isolation, and high relapse rates help to explain the intensity of the 2017 outbreak on the French Guianese side of the border.

Outbreak control measures in French Guiana fostered the use of a specialized malaria team with community health mediators between the months of November and February. This team administered radical treatment to a prodigious fraction of infected individuals. A previous retrospective study in the area did not deploy a similar strategy, and consequently reported less than 5% of infected individuals receiving full treatment [24]. This finding underscores the importance of optimizing response collaborations by integrating community mediators.

The transnational geographic region presented in this study, despite a shared epidemiological setting, possesses two distinct programs for diagnosis and treatment, complicating the implementation of public health programs. Nevertheless, health services on both sides of the border have favorable workforces and technological conditions to positively contribute to successful malaria control. Malaria treatment is free on both sides of the border and “acceptability of interventions” were previously described as “good” in both areas [5]. By countries employ vector control measures, including insecticide-treated bed nets and indoor residual spraying [5]. Brazilian and French Guianese malaria cases appear to be related in space (border area) and time, but also experienced by the same Indigenous populations, notably the Palikur. The Palikur are traditionally a mobile people, commonly traversing the border between French Guiana and the Brazilian villages of Kouméné and Amomi. These two villages are considered the spiritual center for Palikur communities living in Trois-Palétuviers, Philogène or Blondin [32]. Although significant transnational human mobility has been previously documented in this area, more data is necessary in order to better understand local malaria epidemiology [33]. Indigenous health centers are autonomous in Brazil and care is given by and for Indigenous peoples through the National Indian Foundation (FUNAI). Improvements may be needed regarding collaborative efforts between Brazilian health authorities and the FUNAI, in reference to community mobilization, to reach acceptable malaria control metrics in both French Guiana and Brazil. An improved control of malaria in this region requires a global and systematic approach, including partnerships between different disciplines and common commitments among all levels of national institutions.

## Conclusion

The present findings demonstrate an ongoing potential for the increase in malaria cases in an area with otherwise declining numbers. The study area is a transborder region where remote populations challenge malaria control programs. *P. vivax* elimination requires well-coordinated efforts and border-transcendent malaria control methodologies. Novel strategies are vital to achieving elimination, particularly in Indigenous American communities.

## Supplementary information

**Additional file 1: Supplement S1.** Distribution of G6PD deficiency according to the WHO definition of French Guianese participants. **Supplement S2.** Results of clusters for selected localities in the cross-border region between French Guiana and Brazil, January 2017—January 2018. FUNAI: Fundação Nacional do Índio, Brazil; SIVEP—Malária: Sistema de Vigilância Epidemiológica da Malária, Brazil; CDPS: Delocalized Centers for Prevention and Care, Cayenne Hospital, French Guiana; BR: Brazil; GF: French Guiana

**Additional file 2: Supplement S3.** Graphical abstract of the main result of the malaria outbreak investigation, border area between French Guiana and Brazil, 2017

## Data Availability

The datasets generated and analyzed during the present study are not publicly available due to the requirement of special authorization to transfer databases provided by the CNIL. Upon prior CNIL authorization, the datasets can be made available from the corresponding author upon reasonable request.

## Abbreviations

*An. Darlingi*: *Anopheles darlingi*
ANSM: French national agency for medicines and health products safety
BR: Brazil
CDC: Center for disease control and prevention
CDPS: Delocalized centers for prevention and care
CNIL: Commission nationale informatique et libertés
DAAC: Development, animation, accompanying measures, cooperation
DSEI: Special indigenous health district
DNA: Deoxyribo nucleic acid
DNIT: Departamento nacional de infraestruturas de transportes
ETL: Extract, transform and load
FUNAI: Fundação Nacional do Índio, Brazil
G6PD: Glucose-6-Phosphate Dehydrogenase
GF: French Guiana
IBGE: Instituto brasileiro de geografia e estatistica
IGN: Institut national de l’information géographique et forestière, France
IQG: InterQuartile range
LLINs: Long-lasting insecticidal nets
LVC: Lâmina de verificação de Cura
OR: Odds ratio
OSM: OpenStreetMap
PCR: Polymerase chain reaction
*P. falciparum*: *Plasmodium falciparum*
*P. vivax*: *Plasmodium vivax*
RDT: Rapide diagnostic test
**S**IVEP: Sistema de informação de vigilãncia epidemiológica
STG: Saint-Georges de l’Oyapock
WHO: World health organization
