# Supplementary material for: Resurgence risk for malaria, and the characterization of a recent outbreak in an Amazonian border area between French Guiana and Brazil
Source: BMC Infect Dis. 2020 May 26;20:373. doi: 10.1186/s12879-020-05086-4 (PMC7249302; doi:10.1186/s12879-020-05086-4)
Supplement: Supplementary file 1 — Additional file 1: Supplement S1. Distribution of G6PD deficiency according to the WHO definition of French Guianese participants. Supplement S2. Results of clusters for selected localities in the cross-border region between French Guiana and Brazil, January 2017—January 2018. FUNAI: Fundação Nacional do Índio, Brazil; SIVEP—Malária: Sistema de Vigilância Epidemiológica da Malária, Brazil; CDPS: Delocalized Centers for Prevention and Care, Cayenne Hospital, French Guiana; BR: Brazil; GF: French Guiana [file 12879_2020_5086_MOESM1_ESM.docx]

**Supplementary Data :**

**Supplement S1:** Distribution of G6PD deficiency according to WHO definition of French Guianese participants

| **G6PD** | **n** | **% and mean** | **Classification** |
| --- | --- | --- | --- |
|  | 188 | 100 / 12,7 [12,26-13,15] |  |
| *Male* |  |  |  |
| <1U/g Hb | 0 |  | Deficient |
| 1-8 U/g Hb | 10 | 5,3 / 6,51[5,39-7,62] | Intermediate |
| ≥8 U/g Hb | 92 | 48,9 / 12,94 [12,41-13,46] | Normal |
| *Female* |  |  |  |
| <3U/g Hb | 0 |  | Deficient |
| 3-8 U/g Hb | 3 | 1,6 / 6,36 [4,44-8,29] | Intermediate |
| ≥8 U/g Hb | 80 | 42,6 / 13,44 [12,86-14,01] | Normal |

**Supplement S2:** Results of cluster for selected localities on the crossborder area between French Guiana and Brazil, January 2017-January 2018.

| **Locality** | **Cluster** | **Number of Cases** | **country** | **Population** | **Year of population update** | **Pop. Information Source** | **Incidence Rate (case per 100*)** | **Ethnicity** |
| --- | --- | --- | --- | --- | --- | --- | --- | --- |
|  |  |  |  |  |  |  |  |  |
| Benoá | 1 | 31 | BR | 48 | 2015 | FUNAI | 64.6 | Karipuna |
| Amomi | 1 | 13 | BR | 72 | 2015 | FUNAI | 18.1 | Palikur |
|  |  |  |  |  |  |  |  |  |
|  | | | | | | | | |
|  |  |  |  |  |  |  |  |  |
| Manga | 2 | 180 | BR | 817 | 2015 | FUNAI | 22.0 | Karipuna |
| Kumenê | 2 | 167 | BR | 784 | 2015 | FUNAI | 21.3 | Palikur |
| Centro de Oiapoque | 2 | 105 | BR | 1856 | 2015 | SIVEP-M. | 5.6 | Multi-ethnic |
| Planalto | 2 | 73 | BR | 2991 | NA | SIVEP-M. | 2.4 | Multi-ethnic |
| Clevelândia do Norte | 2 | 13 | BR | 709 | NA | SIVEP-M. | 1.8 | Multi-ethnic |
|  |  |  |  |  |  |  |  |  |
|  |  |  |  |  |  |  |  |  |
| Trois Palétuviers | 3 | 72 | GF | 155 | 2016 | CDPS | 46.4 | Palikur (Galibi, Karipuna) |
| Paraíso | 3 | 81 | BR | 1782 | 2015 | SIVEP-M. | 4.5 |  |
| Kunanã | 3 | 30 | BR | 74 | 2015 | FUNAI | 40.5 | Karipuna |
| Ariramba | 3 | 23 | BR | 52 | 2015 | FUNAI | 44.2 | Karipuna |
| Japiim | 3 | 22 | BR | 52 | 2015 | FUNAI | 42.3 | Karipuna |
| Vila do Taparabó | 3 | 22 | BR | 65 | 2015 | SIVEP-Malaria | 33.8 |  |
| Huaha | 3 | 30 | BR | 102 | 2015 | FUNAI | 29.4 | Galibi-Marworno |
| Crikou | 3 | 14 | BR | 51 | 2015 | SIVEP-M. | 27.5 |  |
| Açaizal | 3 | 23 | BR | 112 | 2015 | FUNAI | 20.5 | Karipuna |
| Nova Esperança | 3 | 87 | BR | 2213 | 2015 | SIVEP-M. | 3.9 |  |
| Universidade | 3 | 49 | BR | 1651 | NA | SIVEP-M. | 3.0 |  |
| Nova União | 3 | 49 | BR | 2772 | NA | SIVEP-M. | 1.8 |  |
| Centre de St-Georges | 3 | 22 | GF | 1385 | 2016 | CDPS | 1.6 | Multi-ethnic |
|  |  |  |  |  |  |  |  |  |
|  |  |  |  |  |  |  |  |  |
| Galibi | 4 | 29 | BR | 58 | 2015 | FUNAI | 50.0 | Galiby-Kalinã, Karipuna, Palikur, Galibi-Marworno. |
| Village Blondin | 4 | 9 | GF | 78 | 2016 | CDPS | 11.5 | Palikur |
| Vila Vitória | 4 | 54 | BR | 816 | 2015 | SIVEP-M. | 6.6 | Multi-ethnic |
| Infraero | 4 | 86 | BR | 1452 | NA | SIVEP-M. | 5.9 |  |
| Espírito Santo | 4 | 28 | BR | 505 | 2015 | FUNAI | 5.5 | Karipuna |
| Kumarumã | 4 | 74 | BR | 1749 | 2015 | FUNAI | 4.2 | Galibi-Marworno |
| Maripa | 4 | 16 | GF | 354 | 2016 | CDPS | 4.5 | Multi-ethnic |
| Adimo | 4 | 18 | GF | 510 | 2016 | CDPS | 3.5 | Multi-ethnic |
| Esperance 3 - Savane | 4 | 12 | GF | 855 | 2016 | CDPS | 1.4 | Multi-ethnic |
| Onozo | 4 | 10 | GF | 698 | 2016 | CDPS | 1.4 | Multi-ethnic |
|  |  |  |  |  |  |  |  |  |
|  | **Total** | **1442** |  |  |  |  |  |  |

* Incidence rate: new malaria cases notified for the period from 01/01/2017 to 31/01/2018 divided by the total population of year 2015 and multiplied by 100.

FUNAI: Fundação Nacional do Índio, Brazil; SIVEP-Malária: Sistema de Vigilância Epidemiológica da Malária, Brazil; CDPS: Delocalized Centers for Prevention and Care, Cayenne Hospital, French Guiana; BR: Brazil; GF: French Guiana
