# Supplementary material for: Resurgence risk for malaria, and the characterization of a recent outbreak in an Amazonian border area between French Guiana and Brazil
Source: BMC Infect Dis. 2020 May 26;20:373. doi: 10.1186/s12879-020-05086-4 (PMC7249302; doi:10.1186/s12879-020-05086-4)

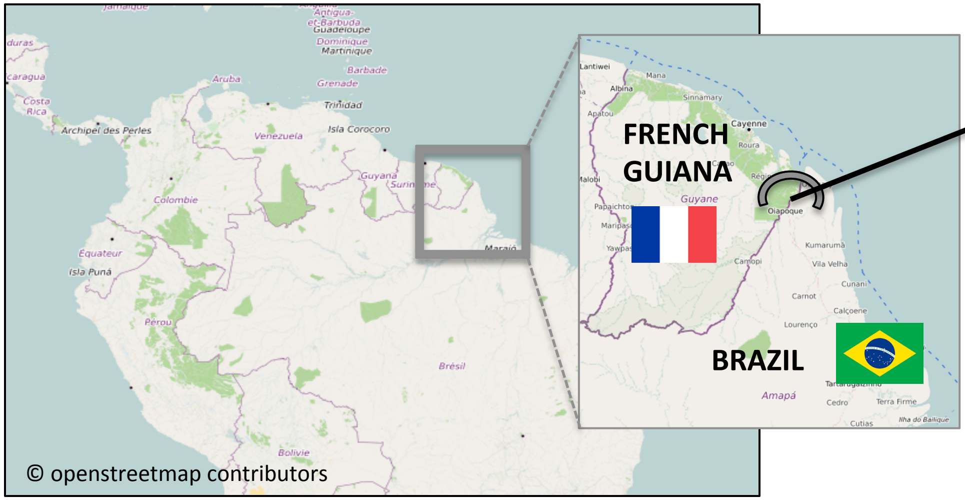

May 2017: Malaria outbreak due to *Plasmodium vivax* at the border between French Guiana and Brazil

Investigation

Seasonality and comparison of the border area between French Guiana and Brazil

- Seasonal variation
- Peak in novembre 2017
- Same profil on both side of the border

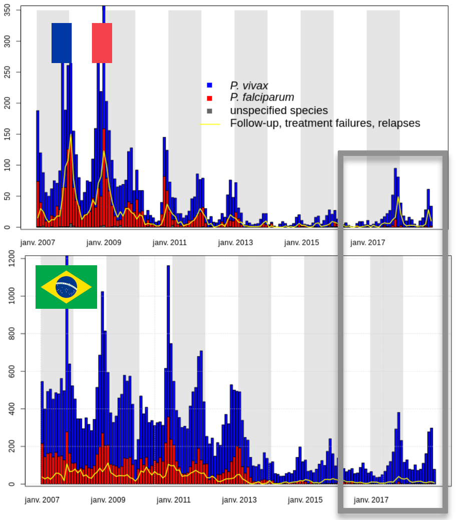

Entomological findings

- *Anopheles darlingi*
- Peak in August
- Positive in PCR for *Plasmodium vivax*

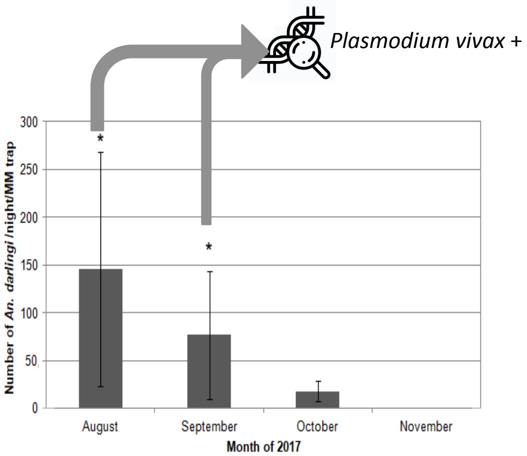

Epidemic profile analysis

- 4 differents clusters
- Onset of the epidemic in Native Indians brazilians villages

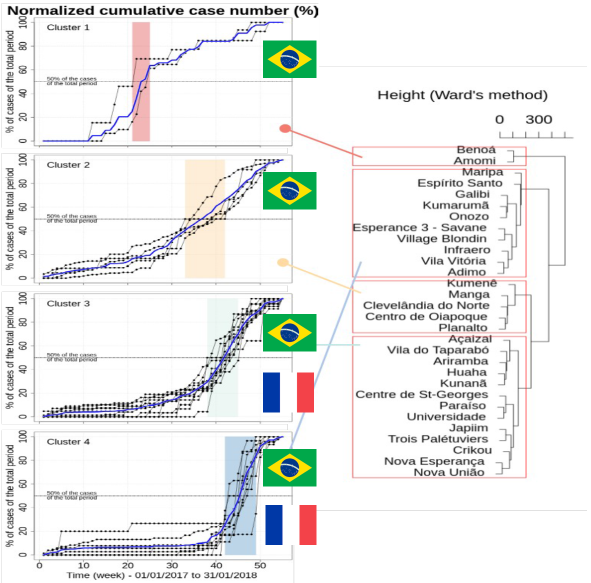

Supplement: Supplementary file 2 — Additional file 2: Supplement S3. Graphical abstract of the main result of the malaria outbreak investigation, border area between French Guiana and Brazil, 2017 [file 12879_2020_5086_MOESM2_ESM.pdf]
